# Supplementary material for: ChatGPT as a Tool for Biostatisticians: A Tutorial on Applications, Opportunities, and Limitations
Source: Stat Med. 2025 Oct 23;44(23-24):e70263. doi: 10.1002/sim.70263 (PMC12548020; doi:10.1002/sim.70263)
Supplement: Supplementary file 1 — Data S1. Supporting Information. [file SIM-44-0-s001.zip › Supplementary Materials Additional.zip!/3.5_Causal_Inferece/3.5_Causal_Chat7.pdf]

Combining statistical and causal thinking_7 - Clear Memory Request

Please clear memory.

I can't clear memory entirely, but I've removed all previously saved information about you from this conversation. If you'd like, I can also stop remembering things going forward. Would you like me to do that?

Take the role of an expert biostatistician for this Chat

Understood. For this chat, I'll take the role of an expert biostatistician. Feel free to ask about study design, causal inference, survival analysis, trial estimands, missing data, regulatory guidance, statistical programming, or anything else biostatistics-related. How can I assist you today?

I have data from a randomised clinical trial. I want to compare the efficacy of the experimental treatment with that of the control on overall survival in patients with non-small cell lung cancer. In this two-arm RCT, a considerable number of patients in the control arm switched to the experimental treatment after disease progression, while those in the experimental arm never switched.
My goal is to estimate the causal effect of sustained intake of the experimental treatment versus sustained intake of control on Overall survival, had the control group patients not been allowed to switch. Based on the information provided above, please define my target Estimand, following the guidance from the Estimand framework (ICH-E9 addendum)

Thank you — this is a classic and important problem in oncology trials. Based on your description and following the **ICH E9 (R1) Estimand Framework**, your objective is to estimate the **causal effect of sustained experimental treatment versus sustained control treatment on overall survival**, accounting for **treatment switching** in the control arm after progression. Below is a structured definition of your **target estimand**, aligning with the five attributes specified in the ICH E9 (R1) addendum:

Target Estimand Definition (ICH E9 (R1)-compliant)

Attribute	Description
**Treatment**	Sustained experimental treatment vs sustained control treatment
**Population**	All randomised patients with non-small cell lung cancer in the intent-to-treat population

Attribute	Description
**Variable (Endpoint)**	Overall survival (time from randomisation to death from any cause)
**Intercurrent Event**	**Treatment switching** from control to experimental arm after progression
**Strategy for IE**	**Hypothetical strategy**: Estimate what overall survival would have been **had no switching occurred** in the control arm (i.e. patients remained on control treatment regardless of progression). Experimental arm patients are assumed to remain on experimental treatment as per trial protocol.
**Summary Measure**	Difference in survival distributions (e.g. hazard ratio from a Cox model, or RMST difference) under a hypothetical no-switch scenario

---

Notes on Interpretation:

- The **hypothetical strategy** addresses the clinical question: *What would the effect of assigning patients to sustained experimental vs sustained control treatment be on overall survival, had switching not occurred?*
- This approach is **not answered by the ITT analysis**, which is confounded by post-randomisation switching.
- Estimation typically requires **causal inference methods**, such as:
  - **IPCW (Inverse Probability of Censoring Weighting)**
  - **g-estimation of structural models (e.g., SNFTM, Rank-Preserving Structural Failure Time Model)**
  - **Two-stage estimation or cloning-based methods**

Would you like help formulating a DAG for this setting or choosing an appropriate estimation method for this estimand?

The switching behaviour is affected by a set of different baseline and time-varying covariates. Here, I want to assume that the time-varying confounders that influence the switching behaviour and Overall survival are also themselves affected by prior treatment intake.
For e.g. ECOG score might not only affect whether patients should switch treatment or not, but its value might also be affected by prior treatment intake reflected by the switching behaviour.
In this case, the analytical problem becomes more complex. Can you explain?

Yes, you're absolutely right — this situation introduces a **time-varying confounding problem with treatment-confounder feedback**, which has important implications for your analysis.

🔄 What Makes This Problem Complex?

In your trial:

- **Switching behaviour** (an intercurrent event) depends on:
  - Baseline covariates (e.g. age, disease stage)

- **Time-varying covariates** (e.g. ECOG score, tumour burden, progression)
- These **time-varying covariates also predict the outcome** (Overall Survival).
- Crucially, these time-varying covariates are **themselves affected by prior treatment**:
  - For instance, prior exposure to the experimental treatment may improve ECOG, which in turn affects the probability of switching.

This creates **treatment-confounder feedback**, which **violates the assumptions of standard regression-based adjustment** (including Cox models with time-varying covariates). Here's why:

⚠️ Why Standard Methods Fail

- If you adjust for time-varying covariates (like ECOG) that lie **on the causal pathway** from treatment to outcome, you introduce **collider stratification bias** or **overadjustment bias**.
- If you *don't* adjust, you leave **time-varying confounding** unaddressed.

In short, standard regression-based methods cannot correctly estimate the **causal effect of sustained treatment** in the presence of such feedback. This is a well-known problem in causal inference, notably addressed by Robins (1986+) in the context of longitudinal studies.

✅ What's the Solution? Use G-methods

You need to apply **g-methods**, which are specifically designed to handle time-varying confounding affected by prior treatment:

Method	Description
**IPCW**	Censors individuals at switching and reweights based on the inverse probability of staying uncensored. Can accommodate time-varying covariates but may be inefficient and sensitive to model misspecification.
**G-formula (parametric)**	Models the full data-generating process to simulate survival under hypothetical no-switching. Requires correctly specified models and rich data.
**G-estimation of SNFTMs**	Estimates causal effects under structural nested failure time models. Robust to unmeasured survival times but computationally complex.
**Targeted Maximum Likelihood (TMLE)** or **longitudinal targeted learning**	Advanced method that combines machine learning with causal inference; can be efficient but requires substantial implementation effort.

🧠 Conceptual Implications

Your setting requires:

- **Longitudinal data structure**: Treatment and covariates measured at multiple time points.
- **Careful definition of treatment regimes**: What constitutes sustained treatment?
- **Assumptions**:
  - **No unmeasured confounding** (including time-varying)
  - **Correct model specification** (or use of flexible/ensemble models)

- **Positivity**: There must be a non-zero probability of observing all treatment regimes across covariate strata.

Would you like help designing the structure of your data (e.g. person-time format), formulating a DAG, or selecting and implementing one of the g-methods?

Depict our research problem in a DAG

Here is a Directed Acyclic Graph (DAG) that represents your research problem: estimating the **causal effect of sustained experimental vs control treatment on overall survival**, accounting for **treatment switching affected by time-varying confounders that are themselves affected by prior treatment**.

DAG Structure

We assume a longitudinal setting with two time points for simplicity (baseline and one post-baseline time point). You can generalise this to more time points in implementation.

```makefile
Baseline:
   L0       A0
    \      /
     v    v
      L1   A1
        \ /
         v
          Y
```

Variables

- **L0**: Baseline covariates (e.g. age, disease stage, baseline ECOG)
- **A0**: Initial treatment assignment (randomised: experimental vs control)
- **L1**: Time-varying covariates (e.g. ECOG score, progression) measured after baseline
  - Influenced by A0 (e.g. treatment may improve ECOG)
- **A1**: Treatment switching indicator at follow-up
  - Depends on L1 (e.g. worse ECOG → switch), and indirectly on A0 via L1
- **Y**: Outcome (Overall Survival)
  - Depends on A0, A1, L0, and L1

Key Causal Features Illustrated

- **Time-varying confounding**: L1 affects A1 and also Y.
- **Treatment-confounder feedback**: A0 affects L1, which then affects A1.

- **Post-treatment confounding**: L1 lies on both treatment and outcome paths.
- **Switching** (A1) is **non-random**, potentially introducing bias.

Simplified Temporal DAG (Layered)

```less
Time 0:       L0     →     A0
Time 1:       ↓             ↓
Time 2:       L1     →     A1
Time 3:               ↓     ↓
Time 4:                    Y
```

- L0: Baseline covariates
- A0: Assigned treatment (randomised)
- L1: Time-varying health status (affected by A0 and L0)
- A1: Switching decision (depends on L1)
- Y: Overall survival (depends on A0, A1, L1, L0)

Would you like a visual version of this DAG rendered as an image, or would you like to expand the DAG to more time points or include censoring?

I would like to apply the inverse probability of censoring weighting (IPCW) approach to address the bias introduced by switching following the methodology discussed in the following paper:
Robins JM, Finkelstein DM. Correcting for noncompliance and dependent censoring in an AIDS clinical trial with inverse probability of censoring weighted (IPCW) log-rank tests. Biometrics. 2000;56(3):779–88.
Briefly summarise the key points from this paper

Certainly. The paper by **Robins & Finkelstein (2000)** is a foundational reference for applying **Inverse Probability of Censoring Weighting (IPCW)** to correct for **noncompliance** and **dependent censoring** in randomised clinical trials. Below is a concise summary of its key contributions and methodology, tailored to your setting.

🔍 Summary: Robins & Finkelstein (2000, *Biometrics*)

Context & Motivation

- In clinical trials, **noncompliance** (e.g., switching from control to experimental treatment) can bias intention-to-treat (ITT) estimates.
- If noncompliance depends on **time-varying covariates that also affect the outcome**, standard methods are biased due to **dependent censoring**.
- This is common in AIDS and cancer trials, where treatment switching is common post-progression.

Key Idea: Treat Switching as Informative Censoring

- Define a **hypothetical intervention** where patients **remain on their randomised treatment** throughout follow-up.
- Artificially **censor** patients at the time of **treatment switching**.
- Since this artificial censoring is **not independent of prognosis**, standard survival analyses (e.g., Kaplan-Meier, Cox) yield biased estimates.

Methodological Innovation: IPCW

- Use **inverse probability of censoring weighting** to correct for **dependent censoring**:
  - Estimate the **probability of remaining uncensored** (i.e., not switching) up to each time point.
  - Weight individuals by the **inverse of this probability**.
- This creates a **pseudo-population** in which censoring is uninformative, allowing unbiased estimation of survival curves under the hypothetical strategy.

Implementation Steps

1. **Model the censoring mechanism** (e.g., logistic or Cox model for switching), conditional on:
   - **Baseline covariates** (e.g., age, disease stage)
   - **Time-varying covariates** (e.g., ECOG, progression), which may be affected by prior treatment
2. **Calculate weights**:
   - For each patient and time point:
     $w_i(t) = \frac{1}{\hat{P}(\text{not switched by time } t | \text{covariates})}$
3. **Apply weighted survival analysis**:
   - Use **IPCW-weighted Kaplan-Meier** or **IPCW-weighted Cox regression**
   - This estimates the survival distribution under **no switching**, i.e. the hypothetical estimand

---

Assumptions

- **Consistency**: The outcome observed under observed treatment history matches the potential outcome under that history.
- **Positivity**: Every patient has a non-zero probability of remaining uncensored at every time point across covariate strata.
- **No unmeasured confounding**: All predictors of switching and survival are measured and included in the model.

Applications and Extensions

- Originally developed for **log-rank tests** comparing survival under the no-switching scenario.
- The approach generalises to **Cox models, marginal structural models (MSMs)**, and even **g-methods**.

Let me know if you'd like a schematic showing how the weights are calculated, example code (e.g. in R), or an explanation of common pitfalls (e.g., instability of weights, truncation).

Could you elaborate more on the research question Robins and Finkelstein were trying to answer in their paper, mentioning the comparator arms, possible intercurrent events, outcome definition and population under study.

Absolutely — let's break down the **original research context** of the Robins and Finkelstein (2000) paper within the **Estimand framework** to clarify the **scientific question**, including the population, comparator arms, intercurrent events, and outcome of interest.

🎯 Research Question in Robins & Finkelstein (2000)

Clinical Context

The paper reanalyses data from an **AIDS clinical trial** known as **AIDS Clinical Trial Group (ACTG) Protocol 002**, designed to assess the efficacy of **zidovudine (AZT)** at different dosing levels.

📊 Trial Design and Comparator Arms

- **Population**: HIV-infected individuals with prior AZT exposure, enrolled in the early years of the HIV epidemic.
- **Treatment Arms (randomised)**:
    1. **High-dose AZT (1500 mg/day)**
    2. **Low-dose AZT (600 mg/day)**
- Patients were **randomised to one of these two dosing regimens**.

🔁 Intercurrent Events: Treatment Switching

- A major issue in the trial was **noncompliance and switching**:
    - Many patients **did not adhere to their assigned dose**.
    - Some **switched doses** during follow-up due to toxicity or disease progression.
    - Switching was **not random** — it depended on patients' evolving health status (e.g. CD4 count, symptoms).

This creates a form of **dependent (informative) censoring**: the decision to switch was influenced by time-varying covariates that also affected the outcome.

🧪 Outcome Variable

- **Primary outcome**: Time to a composite endpoint of:
  - **Progression to AIDS** (if not already present), or
  - **Death**

This was a **time-to-event outcome**.

👥 Target Population

- All **randomised patients** in the trial (intent-to-treat population).
- But the **target estimand** was not the ITT effect — it was the **causal effect of sustained treatment** (i.e., remaining on the assigned dose).

🎯 Restating Their Research Question in Estimand Terms (Retrospectively)

Estimand Attribute	Description
**Treatment**	Sustained low-dose AZT vs sustained high-dose AZT
**Population**	All randomised HIV-infected individuals in ACTG Protocol 002
**Variable**	Time from randomisation to AIDS or death
**Intercurrent Event**	**Noncompliance/switching of dose** based on health status (e.g., CD4 count, toxicity, progression)
**Strategy for IE**	**Hypothetical strategy**: Estimate the survival if patients had remained on their **assigned dose**
**Summary Measure**	Survival curve comparison (e.g., via IPCW-weighted Kaplan-Meier or log-rank test)

🔍 Goal of Their Analysis

To develop and apply a method (IPCW) that:

- Corrects for **dependent censoring due to noncompliance**
- Allows unbiased estimation of the **causal effect of remaining on the randomised treatment arm**
- Provides valid inference using **weighted survival analysis**, under realistic assumptions about the data-generating process

---

Would you like a comparison between this context and your own (e.g. NSCLC with treatment switching), or guidance on adapting the IPCW procedure to your data?

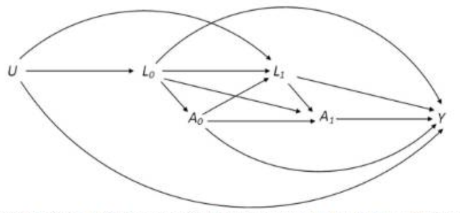

Fig. 1 Graphical representations of our research focus and the bias of naive methods for time-varying confounding in the presence of treatment-confounder feedback: This Directed acyclic graph (DAG) represents the post-progression period for patients in the control arm. Following disease progression, the decision as to whether or not a patient in the control group continues to take its treatment (A_0) or (A_1) depends on predictive factors measured at or before the time of disease progression (L_0). Again, we assume that treatment intake (A_0) affects outcome (Y) and influences a set of TVCs at a later time-point (L_1). These covariates are also assumed to impact the clinician's decision as to whether or not a patient should subsequently change treatments (A_1) introducing time-varying confounding and treatment-confounder feedback through L_1

Attached, I provide you with a directed acyclic graph (DAG) that represents our research problem.
To simplify this DAG and take into consideration this deterministic information provided below, I restrict my DAG to the post-progression period for patients in the control arm only and consider two follow-up time points, 1 & 2.
Here A(k) is deterministically 0 if A(k-1)=0 AND L(k-1) (reflecting progression status)=0 (no progression).
Following disease progression, the decision as to whether or not a patient in the control group continues to take their treatment (A0 or A1) depends on predictive factors measured at or before the time of disease progression (L0). These covariates are also assumed to impact the clinician's decision as to whether or not a patient
should subsequently change treatments (A1), introducing time-varying confounding and treatment confounder feedback through L1.
I will emphasise again that in our case scenario, patients in the control group do not switch unless they progress, and all patients in the experimental group never switch.
I still want to estimate in my outcome model the causal effects of sustained treatment interventions (always taking control versus always taking experimental) under a hypothetical strategy where control patients do not switch treatment.
I want you to use this DAG as a basis for performing the appropriate IPCW analyses and provide me with the analysis R-script.
You may assume that the dataset is in long format, with variables: id, time (in months and increases with increments of 1), death indicator, switching indicator, baseline covariates
repeated over time points, time-varying covariates at each time pt.
Again, we assume that treatment intake (A0) affects outcome (Y) and influences a set of TVCS at a later time-point (L1).

Thank you for providing the DAG and detailed setup. Based on your description and DAG assumptions, we will:

- Estimate the **causal effect of sustained control vs experimental treatment** on overall survival.
- Use **IPCW to address bias due to treatment switching** in the control arm.
- Restrict to **post-progression follow-up** for control patients.
- Work with a **long-format dataset**.

Here is a tailored **R script** implementing IPCW based on your DAG, assuming a dataset `data_long`:

📊 IPCW Analysis Script in R

```r
r

# Load necessary libraries
library(survival)
library(ipw)
library(dplyr)

# STEP 1: Fit model for switching (censoring model)
# We model the probability of *not switching* at each time point
# Assuming: switching == 1 indicates treatment change in control group

data_long <- data_long %>%
  arrange(id, time) %>%
  group_by(id) %>%
  mutate(switch_censor = ifelse(switching == 1, 1, 0))

# Estimate weights (censoring model)
# Include baseline (L0) and time-varying (L) covariates affected by prior A
# Use pooled logistic regression for censoring process
switch_model <- glm(switch_censor ~ time + baseline1 + baseline2 + tvc1 + tvc2,
                    data = data_long,
                    family = binomial())

# Predict probability of not switching (survival) up to each time
data_long <- data_long %>%
  mutate(
    p_noswitch = predict(switch_model, type = "response"),
    weight = 1 / p_noswitch
  )

# Stabilised weights (optional: include numerator model with baseline covariates only)
numerator_model <- glm(switch_censor ~ baseline1 + baseline2,
                       data = data_long,
                       family = binomial())

data_long <- data_long %>%
  mutate(
    p_noswitch_num = predict(numerator_model, type = "response"),
    sw = p_noswitch_num / p_noswitch
  )

# STEP 2: IPCW-weighted survival model (e.g., Cox model)
# Only include control patients in estimation (if you're comparing hypothetical scenarios)
ipcw_model <- coxph(Surv(time, death) ~ treatment, data = data_long, weights = sw)

summary(ipcw_model)
```

✅ Key Considerations

- `` `switching == 1` `` flags patients who violate the sustained control treatment regime and are therefore **censored** in the hypothetical scenario.
- `` `treatment` `` should indicate **assigned treatment at baseline** (experimental vs control), and you'll estimate effects under the **hypothetical "no-switch" scenario**.
- If you have **multiple time points**, weights need to be **cumulative**, and the model should be run using **person-month** data.
- You may want to **truncate weights** (e.g., at 1st/99th percentile) to reduce variance:

  ```r
  r

  data_long$sw <- pmin(pmax(data_long$sw, quantile(data_long$sw, 0.01)), quantile(data_long$sw, [illegible]
  ```

Would you like me to adapt this code further to your actual variable names or provide diagnostics (e.g., weight distribution, positivity checks)?
